# A review on molecular detection techniques of white spot syndrome virus: Perspectives of problems and solutions in shrimp farming

**DOI:** 10.1002/vms3.979

**Published:** 2022-10-25

**Authors:** Sk Injamamul Islam, Moslema Jahan Mou, Saloa Sanjida, Sarower Mahfuj

**Affiliations:** ^1^ Department of Fisheries and Marine Bioscience, Faculty of Biological Science Jashore University of Science and Technology Jashore Bangladesh; ^2^ Department of Genetic Engineering and Biotechnology Faculty of Life and Earth Science University of Rajshahi Rajshahi Bangladesh; ^3^ Department of Environmental Science and Technology Faculty of Applied Science and Technology Jashore University of Science and Technology Jashore Bangladesh

**Keywords:** biosecurity, diagnosis, economy, pathogenicity, transmission, WSSV

## Abstract

This review aims to provide an update on the current scientific understanding of various aspects of White Spot Syndrome Virus (WSSV) formation, diagnostic procedures, transmission, ecological effects, pathophysiology and management strategies. In terms of production and financial benefits, the WSSV has been the most virulent in shrimp and several other crustacean sectors around the globe. It spreads vertically from diseased broodstock to post‐larvae and horizontally by cannibalism, invertebrate vectors, freshwater and sediments. In the transfer of white spot disease (WSD) in newly stocked ponds, the survivability of WSSV in sediment is the most important variable. In typical cultural conditions, it is a highly infectious pathogen capable of inflicting total death within 3–10 days after an outbreak. Some of the current biosecurity strategies used to keep diseases out of shrimp ponds such as pond water disinfection, quarantine of new stocks before stocking and broader usage of specific pathogen‐free shrimp. The sequencing and characterisation of various WSSV strains have provided details about pathogen biology, pathogenicity and disease. To develop successful control methods, knowledge of these characteristics is essential. In several shrimp‐producing countries in Asia and the Americas, the infections produced by the WSSV have had disastrous socio‐economic consequences. As a result of international trade or migration of diseased species, the World Animal Health Organization recognised several illnesses as posing a substantial hazard to farmed shrimp. WSD is receiving much scientific research due to the potential economic effects of the virus. Research is now being done to understand better the molecular biology and pathophysiology of WSSV, as well as how to treat and prevent the virus. However, further study should be conducted in countries with more resilient host species to understand their role in mitigating disease impacts since these revelations may aid in developing a WSD treatment.

## Abstract 2

The goal of this paper is to focus the molecular techniques reported till now to detect WSSV on shrimp more conveniently. Moreover, the implication of biosecurity measures to control and prevent WSSV on shrimp also mentioned along with the latest prevention application.

## 1 INTRODUCTION

The pathogen that causes white spot disease (WSD) in cultured penaeid shrimp is the White Spot Syndrome Virus (WSSV). This virus is a solitary individual from the Nimaviridae virus family, which was recently discovered (Pradeep et al., 2012). WSSV is a big double‐stranded DNA baculovirus, and infection with the WSSV pathogen in shrimp farming can reach a 100% death rate in 3–10 days (Oakey et al., 2019). The white dots on the carapace of shrimp infected with WSSV are immediately identifiable (Saravanan et al., 2017). On the other hand, the initial clinical signs arise after the animal has been infected and is near death. As a result, diagnosis methods have evolved from morphological‐based characterisation utilising electron microscopy (EM) to exceedingly effective immunological and molecular methods that previously could only recognise the infection in asymptomatic carriers (Curry et al., 2006b). White spots, then again, may not appear in all diseases or all species (Md Rahman, 2021).

WSSV was initially discovered in farmed *Penaeus japonicus* in Japan; however, it is believed that it was brought with live diseased post‐larvae (PL) from Fujian Province, Mainland China, in 1992 (Jiang et al., 2017; W.‐B. Zhan et al., 1998). Subsequently, the virus soon spread through most of Asia's shrimp‐producing countries, most likely due to infected broodstock and PL of *Penaeus monodon*. The virus entered the Pacific Ocean in 1995, possibly due to the transfer of infected PL, as it was discovered in Texas, North America and South Carolina, USA, 1 year later (Rosenberg, 2000; Schalie, 1999). The virus did not make it to the Latin American pacific shrimp farming countries until 1999, when it wreaked havoc in Ecuador, Peru and Mexico. Shrimp farming has been conducted on a small scale in coastal Southeast Asia for generations when farmers grew wild shrimp as a side crop in tidal ponds (Kungvankij and Fi 1984; J. Lotz, 1997).

Since the disease's occurrence, the economic losses associated with the disease condition to the shrimp sector have been predicted to be eight to fifteen billion dollars worldwide (D. Lightner et al., 2012; Shinn et al., 2018). The financial losses had already been growing at a rate of USD 1 billion every year (Dashtiannasab, 2020; Escobedo‐Bonilla et al., 2008; FAO, 2014). WSSV was blamed in China for 80% of farmed shrimp production losses (Briggs et al., 2004; Salehi, 2021) and the influence of WSSV on Ecuador's farmed shrimp output was likewise severe (Lucien‐Brun, 2017). The transmission of WSSV to several other shrimp‐producing nations threatens the industry's development. As a result, WSD jeopardises shrimp businesses such as farms, feed manufacturing, processing factories and hatcheries, increasing employment. Even though WSD has been causing havoc in shrimp farms around the world since 1991, surprisingly, the illness is still not within grip at the farm level (Bondad‐Reantaso and Subasinghe 2001; Flegel, 2009; D. Lightner, 2011; D. V. Lightner and Redman, 2010; S Salim, 2012; Walker and Mohan, 2009).

WSSV infection and disease can affect a wide variety of crustaceans. These hosts’ natural populations can also serve as a reservoir for the infectious agent. Given the severity of WSD in captive crustaceans, it is hardly unexpected that much effort has gone into figuring out the disease's fundamental causes and finding potential remedies for disease control or mitigation (Clay, 1997; C. F. Lo, 2014; Nguyen et al., 2009; Sánchez‐Paz, 2010). Cultured shrimp have a maximum life span of 4–5 months. Shrimp must tolerate increasingly varied circumstances, including temperature and salinity fluctuations, during the culture stage (Broom, 2009; Joseph et al., 2013). Excessive salinities are a significant environmental stressor that may increase shrimp susceptibility to viral diseases (Abad‐Rosales et al., 2018; Ramos‐Carreño et al., 2014). Additionally, genetic markers resulting from mutations might be a possible cause of the molecular diagnostic error due to interference of PCR primer binding, which could result in non‐specific PCR products or incorrect negative PCR findings, limiting the utility of PCR‐based therapeutic agents (Mendoza‐Cano and Sánchez‐Paz, 2013a; X. Pan et al., 2016). Despite the hurdles posed by its unique genotypes and infection process, which differs significantly from that of other viruses, much progress has been made in WSSV research (L. Li et al., 2006; Witteveldt et al., 2004). Researchers revealed the complete genome sequencing of the WSSV (2000) (van Hulten et al., 2001). The structural proteins of the virion, the genes that control their production and their function in the host species have been identified (Chaivisuthangkura et al., 2010; Sánchez‐Paz, 2010; Wan et al., 2008). Other research has looked into the virus's transfer to the host body from one species to another, as well as the role of physicochemical qualities of water in infection (Selvam et al., 2012). WSD, like most other viral infections, cannot be treated at this time; hence, it is recommended that the virus be kept out of shrimp farms (Menasveta, 2002). Several biosecurity strategies, antibiotics and vaccinations have been investigated to increase shrimp behaviour to WSSV infection (Dey et al., 2019; Feng et al., 2017). Specific pathogen‐free (SPF) shrimp broodstock domestication, insignificant water exchange and culture pond dry‐out procedure after each harvesting period are different biosecurity methods that have been used to avoid the introduction of WSSV (D. V. Lightner, 2005; Tendencia et al., 2011).

Most WSSV reviews have focused on advanced genetic analyses and biosecurity measures used in WSD maintenance (Bir et al., 2017; Dey et al., 2019; B. Dieu et al., 2021; Jana et al., 2018). On the other hand, shrimp farming practices are evolving, and recently, certain innovative technologies have been applied to raise yields and better WSD monitoring. New experimental molecular detection methodologies, the impact of WSSV in shrimp farming, development of SPF shrimp, the advantage of specific pathogen‐resistant (SPR) shrimp, the function of WSSV viability owing to physio‐chemical changes in pond sediment and their potentiality in dealing with the WSSV scourge in shrimp production are all highlighted in this study.

## 2 WSSV GENOME

The genome of WSSV is a circular dsDNA molecule that is quite possibly the most comprehensively sequenced viral genome (B. Dieu, 2010; Parrilla‐Taylor et al., 2018; Vlak et al., 2004). Depending on the viral isolate, the genome size varies. Thailand, China and Taiwan had genome sizes of 292,967, 305,107 and 307,287 bp, respectively, according to three total WSSV variants (accession numbers AF369029, AF332093 and AF440570) (Kang et al., 2009; Megahed et al., 2019; Sablok et al., 2012). According to nucleotide sequence analyses, about 185 open reading frames (ORFs) of 50 amino acids or more are encoded by the WSSV genome. After successfully confirming the WSSV genomic DNA sequence, researchers focus on quantitatively studying genetic products, specifically the role of viral envelope proteins. A small number of functional and DNA metabolic genes have been found and characterised (de‐la‐Re‐Vega et al., 2011; Guevara‐Hernandez et al., 2012).

## 3 WSSV VIRION PROTEINS

A virion is a sophisticated assemblage of macromolecules adapted to protecting and delivering viral DNA. Its structural proteins have been highly significant, as they are the primary particles to interface with the host, performing cell targeting functions and stimulating most defences (Tsai et al., 2004). Structural proteins and their genetic sequence must be characterised to determine a virus's taxonomic categorisation. The structural function and collaboration of the virion proteins of WSSV may clarify the virus's unusual morphological attributes. In response, diagnostic assays focused upon a few of these cellular components may be created (Yang et al., 2002). Various WSSV proteins have been recognised. Non‐structural proteins have been distinguished as being engaged with transcriptional regulation (VP9) (Y. Liu et al., 2006), viral multiplication (WSV 021) as well as DNA replication regulation (WSV 477). The envelope contains 21 proteins, the nucleocapsid contains 10 proteins and the tegument contains five proteins. The viral genome replication, virus particle production and cell function inhibition depend on non‐structural proteins found in the WSSV genome. As a result, these proteins are promising candidates for medical research and vaccine development. Western blotting, mass spectrometry and immunoelectron microscopy were used to identify VP9, a complete WSSV protein encoded by ORF115 in diseased *P. monodon* shrimp gills and stomach as a novel, non‐structural protein. Even though the specific physiological role of VP9 is unknown, investigations have shown that it is a common protein in host tissue which is WSSV (Y. Liu et al., 2006). VP9 has a DNA binding fold containing Zinc ion active site that is distinct according to structural investigations using X‐rays and NMR (Banci et al., 2002; Rosenzweig et al., 1999). These findings suggest that VP9 may have a role in WSSV transcription. Because envelope proteins are commonly involved in viral entry, assembly and budding, they are especially critical for enveloped viruses (Chazal & Gerlier, 2003). A cell attachment motif is present in the envelope proteins VP31, VP110 and VP281. This motif is involved in the viral entrance Single‐cell attachment motif can also be found in the tegument proteins VP36A and the nucleocapsid proteins VP664 (Leu et al., 2005;Tsai et al., 2004) and VP136 A (C. Huang et al., 2002; Tsai et al., 2004; X. Xie et al., 2006). Other proteins discovered in the envelope include VP28 (Yoganandhan et al., 2006), VP39B, VP31A, VP41B, VP51A, VP51B, VP68, VP124, VP150, VP187, VP281, VP292 and a collagen‐like protein (L. Li et al., 2005), whereas the proteins VP35 (L.‐L. Chen et al. 2002), VP466 (C. Huang et al., 2002), VP15, VP51 and VP76 (C. Wu and Yang, 2006) are present in the nucleocapsid (Yang et al., 2002). VP466 is one of these proteins that has been discovered to play a crucial function in viral penetration (W. Wu et al., 2005). P466 is a glutathione S‐transferase fusion protein denoted by ORF151 and is one of the latencies associated with the genes in WSSV (X. Xie et al., 2006). ORF112 encodes VP76, and it is implicated in WSSV infection. It involves the conserved domain of Class I cytokine receptors (Yang et al., 2002). ORF112 is a 2025‐nt quality that codes for a 675‐aa protein with a molecular mass of 76 kDa (R. Huang et al., 2005). ORF112 has a signal peptide and multiple glycosylation sites but no transmembrane domain, according to phylogenetic analyses. This suggests that WSSV has designed techniques to circumvent the host's defence mechanism (Table 1).

**TABLE 1:** White spot syndrome virus (WSSV) proteins that have been identified so far

Protein names	Amino acid residues size	Apparent size (kDa)	Putative function	Location in WSSV virion	References
VP9	79	9	Transcriptional	Non‐structural	Li et al. (2005)
VP11	433	11	–	Not determined	(Tsai et al., 2004)
VP12A (VP95)	95	11	Structural	Tegument	(X. Xie et al., 2006)
VP12B (VP68)	68	7	Structural	Envelope	(C. Wu and Yang 2006)
VP13A	100	13	Energy metabolism	Not determined	(Tsai et al., 2004)
VP13B (VP16)	117	13	Structural	Envelope	(X. Xie et al., 2006)
VP14	97	11	Structural	Envelope	(X. Xie et al., 2006)
VP15	80	15	DNA binding protein	Nucleocapsid/core	(Hulten and Vlak 2021)
VP19	121	19	Structural	Envelope	(Tsai et al., 2004)
WSV021	200	23	Regulation virus replication	Non‐structural1	(Zhu et al., 2007)
VP22 (VP184)	891	100	–	Not determined	(Tsai et al., 2004)
VP24 (VP208)	208	24	Structural	Nucleocapsid	(M. C. W. Hulten et al., 2000)
VP26	204	26	Structural	Tegument	(Tsai et al., 2004)
VP28	204	28	Structural	Envelope	(M. C. W. Hulten et al., 2000)
VP31	261	31	Cell attachment	Envelope	(X. Xie et al., 2006)
VP32	278	32	Structural	Envelope	(L. Li et al., 2005)
VP33 (VP 281)	281	32	Cell attachment	Envelope	(L.‐L. Chen et al., 2002)
VP35	228	26	Structural	Nucleocapsid	(X. Xie et al., 2006)
VP36A	297	36	Cell attachment	Tegument	(C. Huang et al., 2002)
VP38A	309	35	Structural	Envelope	(Tsai et al., 2004)
VP38B	321	38	Endonuclease	Not determined	(X. Xie et al., 2006)
VP39A	419	39	Structural	Tegument	(Tsai et al., 2004)
VP39B	283	32	Structural	Envelope	(X. Xie et al., 2006)
VP41A (VP292)	292	33	Structural	Envelope	(X. Xie et al., 2006)
VP41B (VP300)	300	34	Structural	Envelope	(X. Xie et al., 2006)
VP51A	486	51	Structural	Envelope	(Edgerton, 2004)
VP51B (VP384)	384	46	Structural	Envelope	(X. Xie et al., 2006)
VP51C (VP466)	466	50	Structural	Nucleocapsid	(Tsai et al., 2004)
VP53A (VP150)	1301	144	Structural	Envelope	(X. Xie et al., 2006)
VP53B	968	53	Signal transduction pathway	Not determined	(Tsai et al., 2004; X. Xie et al., 2006)
VP53C	489	53	–	Not determined	(X. Xie et al., 2006)
VP55 (VP448)	448	55	–	Not determined	(Tsai et al., 2004)
VP60A (VP56)	465	60	Structural	Envelope	(Ramırez‐Douriet et al., 2005)
VP60B (VP544)	544	60	Adenovirus fibre‐like protein	Nucleocapsid	(Tsai et al., 2004)
VP75	786	75	Structural	Nucleocapsid	(X. Xie et al., 2006)
VP76 (VP73)	675	76	Class 1 cytokine receptor	Nucleocapsid	(X. Xie et al., 2006)
VP90	856	96	Structural	Envelope	(Tsai et al., 2004)
VP95	800	89	Structural	Tegument	(X. Xie and Yang 2006)
VP110	972	110	Cell attachment	Envelope	(Zhang et al., 2004)
VP124	1194	124	Structural	Envelope	(Tsai et al., 2004)
VP136A	1219	136	Cell attachment	Nucleocapsid	(Tsai et al., 2004)
VP136B	1243	136	**–**	Not determined	(Tsai et al., 2004)
VP180 (VP16840)	1684	169	Collagen‐like protein	Envelope	(Tsai et al., 2004)
VP187	1606	174	Structural	Envelope	(Tsai et al., 2004)
VP190	1565	174	Structural	Nucleocapsid	(X. Xie and Yang 2006)
WSV477	208	30	DNA replication	Non‐structural	(Han and Zhang, 2006; Shi et al., 2008)
VP664	6077	664	Cell attachment	Nucleocapsid	(Tsai et al., 2004)
VP800	800	90	**–**	Not determined	(Tsai et al., 2004)

## 4 MORPHOLOGY AND STRUCTURE OF WSSV

**FIGURE 1:**
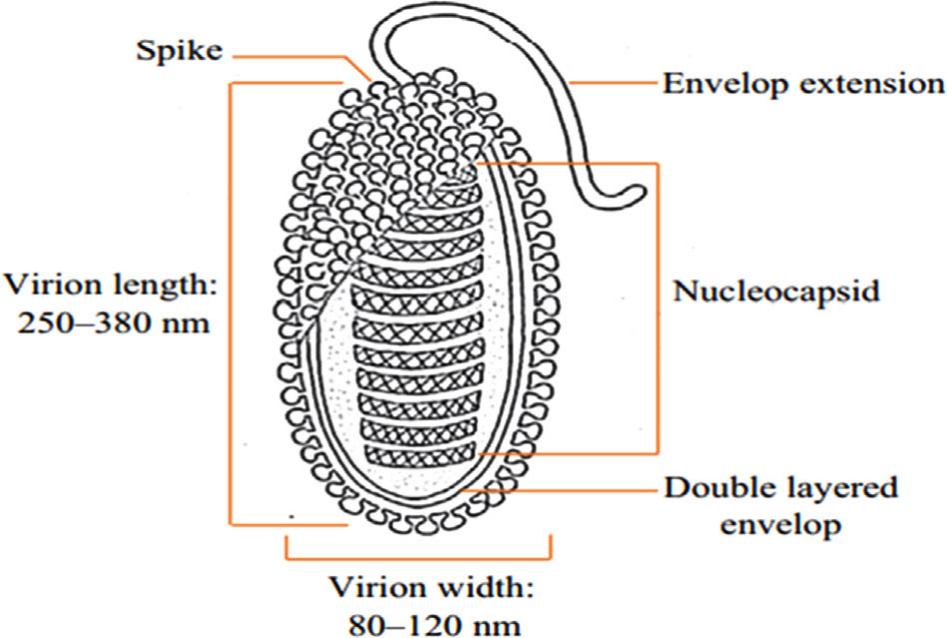
Structure of WSSV

**FIGURE 2:**
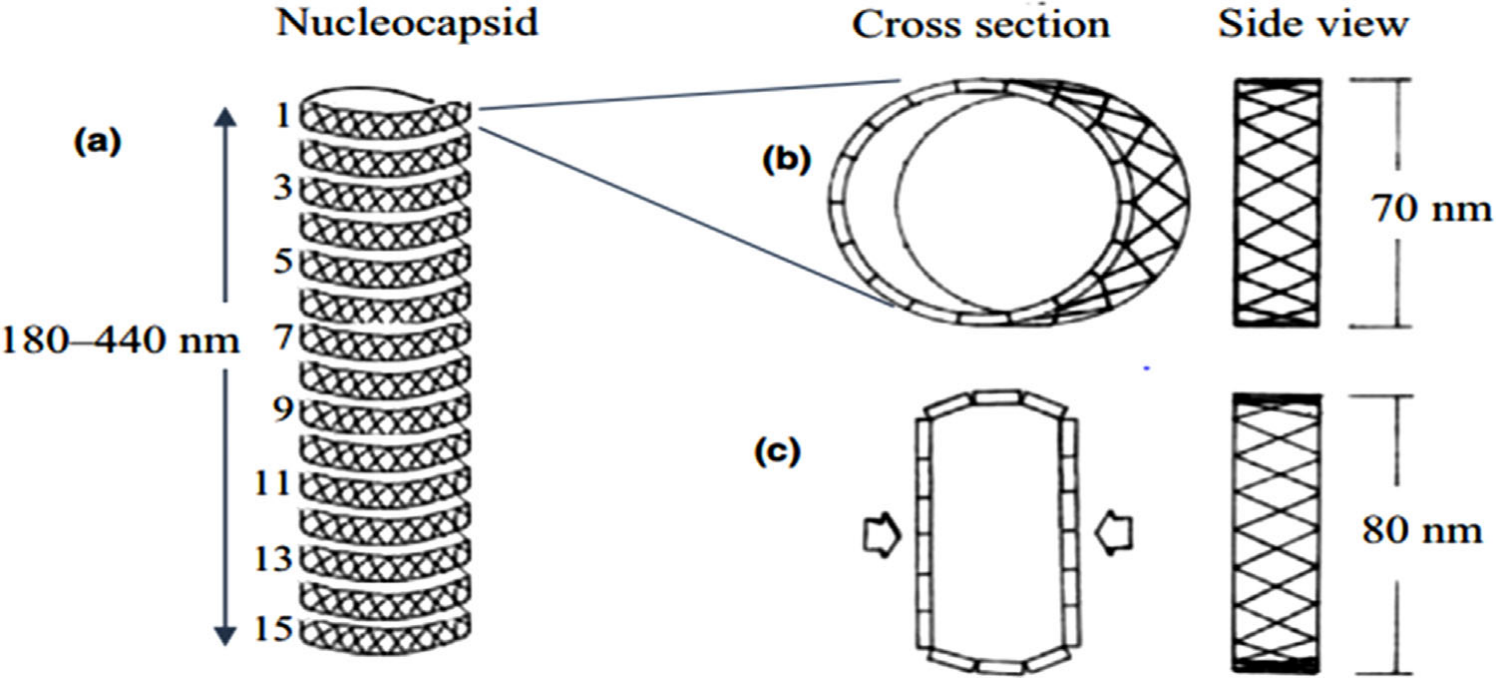
The structure of a WSSV nucleocapsid has been drawn based on the information supplied in the morphological section. (a) The WSSV nucleocapsid, which has 15 prominent vertical helices, was examined using electron microscopy. (b) A cross section of a single nucleocapsid helices with a diameter of 70 nm (side view). (c) An empty nucleocapsid (cross section) having an 80 nm diameter (side view). The nucleocapsid's cross‐hatched structure is a distinctive WSSV identifying characteristic (Verbruggen et al., 2016).

**FIGURE 3:**
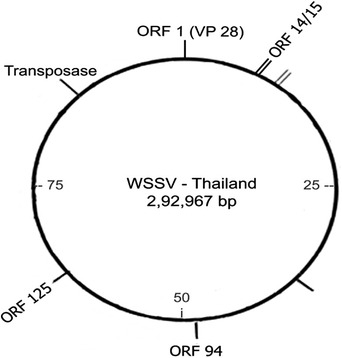
Some of the ORFs and the dominant envelope protein coding gene VP28 are shown in the genome sequence of WSSV from Thailand (WSSV‐TH).

WSSV has a diameter of 80–120nm and a length of 250–380nm (Durand et al., 1996). The virions contain a distinct flagella‐like appendage at one point and are rod‐shaped to elliptical (Walker and Mohan, 2009). At least 45 structural proteins are found in virions, which are organised into three morphologically different layers (Tsai et al., 2004). The virions proliferate without producing occlusion bodies inside the nuclei of infected cells. The WSSV virus attacks ectodermal and mesodermal tissues (Jesudhasan et al., 2000). The nucleocapsid proteins include a basic DNA binding protein (VP15) and a big protein (VP664) that make up the stacking ring components (Verbruggen et al., 2016; Witteveldt et al., 2005). The viral envelope does have a cellular structure that is 6–7 nm thick with 35 distinct proteins, the most numerous of which are VP28 and VP26, which account for around 60% of the envelope (Hulten, 2001; Sánchez‐Paz, 2010) (Figures 1, 2, 3).

## 5 ORGANS OF TARGET AND MECHANISMS OF TRANSMISSION

Ectodermal and mesodermal targets for WSSV transmission include the skin, gill, antennal gland, hindgut, foregut, gonads, haematopoietic cells, lymphoid organ and cells linked with the nervous system (A and R, 2010; Kv et al., 1999). Endodermal organ epithelial cells, such as those in the hepatopancreas, anterior and posterior midgut caeca and midgut trunk, are resistant to WSSV infection (Sahul Hameed et al., 1998). The epithelia of the stomach, gills and integument may be extensively injured in the late stages of illness. This could result in several organ malfunctions and death in the worst‐case scenario. The portals by which WSSV enters the shrimp have yet to be discovered. Variations in entrance sites have been discovered in studies. However, in situ hybridisation demonstrated that the most common locations of WSSV reproduction in early juvenile *P. monodon* were subcuticular epithelial cells of the stomach and cells in the gills, integument and connective tissue of the hepatopancreas (upon os challenge using WSSV damaged tissue) (P. S. Chang et al., 1996). Further studies in *Marsupenaeus japonicus* revealed that epithelium in the midgut trunk might serve as a transitory site for WSSV replication, enabling the virus to circumvent the surrounding basal lamina (Leonardo et al., 2005; S. Xie et al., 2015). WSSV challenge by immersion, on the other hand, revealed that at late stages of infection, haemocytes moving to the gills and midgut were WSSV negative (Arts et al., 2007). Cells in the antennal glands, as well as epithelial cells in the foregut and cells in the gills, were revealed to be foci of WSSV replication when shrimp were per os challenged with viral proteins (with a high infectious dosage) (Escobedo‐Bonilla et al., 2008).

## 6 DIAGNOSTIC METHODS OF WSSV

Several diagnostic methods have been developed to identify WSSV. Gross observation, histopathological techniques (Reyes‐López et al., 2009), in situ hybridisation (D. Lightner et al., 2012), immunological methods such as nitrocellose‐enzyme immunoblot (Nadala et al., 1998) and Western blot techniques (Durand et al., 1997) and polymerase chain reaction (PCR)‐based approaches have lately become more accessible, sensitive and reliable (T. Kim et al., 1998). The sensitivity of these techniques varies depending on whether they are DNA‐based. External appendages can be used to detect preliminary WSSV. The inside surface of the carapace of severely infected shrimp often has a loose cuticle with white spots ranging from 0.5 to 2.0mm in diameter (Hossain et al., 2015). The cuticular epidermis deposits calcium salts abnormally, resulting in this white spot. The expansion of the cuticular chromatophores gave shrimp a pink to reddish‐brown coloration in many cases.

### 6.1 Histopathology

Davidson's fixative should be injected into live moribund prawns from a probable WSSV epidemic. This extremely acidic fixative decalcifies the tissue, making it easier to process and section. Traditional fixative solutions, including 10% neutral buffered formalin, do not decalcify or penetrate the shell, resulting in poor fixing and cutting difficulty. Tissues can be repaired indefinitely without losing quality if the diagnosis is based on histology. However, after 24–48 h, specimens for ISH should be changed from Davidson's fixative to 70% ethyl alcohol, or they will be inappropriate for ISH. The specimens are embedded in paraffin wax and processed using standard procedures after fixing them. WSSV infection is indicated by the inclusion of bodies in target tissues(Reyes‐López et al., 2009).

### 6.2 Electron microscopy

Generally, EM seems to be the first line of defence regarding viral identification (C. S. Goldsmith and S. E. Miller, 2009). With several exceptions (mimiviruses, poxviruses and some iridoviruses), optical microscope decision is insufficient to identify viruses; however, transmission electron microscopy (TEM) resolution is a thousand times greater than optical microscope resolution, enabling rapid identification of microscopic viruses. TEM, on the other hand, can only identify the virus up to the family or genus level; however, this is a rapid procedure (Curry et al., 2006a). Even though the detection limit (DL) of viruses was originally 106 viruses per ml when using TEM alone, this limit has now been reduced to 102 viruses per ml when employing the filtering approach (Laue and Bannert, 2010). There are several benefits to utilising EM, including remembering the capacity to see everything, for example (Laue & Bannert 2010), the norm for the distinguishing proof of new viral diseases (Biel and Gelderblom, 1999), identification of double infections involving several viruses (C. Goldsmith and S. Miller, 2009; Kennedy, 2005) and the capacity to rapidly recognise morphological differences (C. Goldsmith and S. Miller, 2009; Kennedy, 2005). As a consequence, EM can be coupled with multiple approaches and employed as a cutting‐edge method (Hazelton and Gelderblom, 2003).

### 6.3 Immunological techniques

Employing western blot analysis, dot blotting and immunohistochemistry, specific monoclonal antibodies (MAbs) have been shown to discriminate WSSV in shrimp while avoiding cross‐reactions with other viral or shrimp proteins (Vaniksampanna et al., 2016); Specific MAbs, on the other hand, can bind to the WSSV target protein. For instance, MAb targeting VP28 could only bind to WSSV VP28 and not to any other viral or shrimp antigens (Marappan et al., 2006). Compared with a single‐step PCR, these immunological approaches have a higher diagnostic sensitivity (100%) (PCR). Dot blotting, a well‐known antibody‐based protein detection approach, on the other hand, is reported to require sufficiently large viral loads for detection (Sithigorngul et al., 2011). Two MAb cocktails were 100 times more accurate than one‐step PCR and approximately equivalent to two‐step PCR detecting WSSV, focusing on its VP28 envelop protein. Additionally, when a MAb cocktail was used instead of a single MAb, the DL of WSSV was increased by two to four times (Inchara et al. 2018). In several antibody‐based tests, scientists recommended employing a mix of VP19 and VP28 (virion proteins) targeted MAbs to boost the specificity of WSSV diagnosis in shrimp (Chaivisuthangkura et al., 2010). An immunosensor has been designed to detect WSSV in shrimp pond water quantitatively. That immunosensor was particularly sensitive for testing WSSV in the linear range 1.6 × 10(1) – 1.6 × 10(6) copies/μl. Moreover, the immunosensor was reusable up to 37 times and was straightforward (Waiyapoka et al., 2015). Another study used gold nanoparticles coupled to a polyclonal anti‐VP28 antibody, lateral flow immunoassay (LFIA) was shown to be a rapid and convenient approach for detecting WSSV in under 20 min (Underwood et al., 2013). Different shrimp viruses, such as infectious hypodermal and hematopoietic necrosis virus (IHHNV), hepatopancreatic parvovirus (HPV) and monodon baculovirus (MBV), showed no cross‐reactivity with the LFIA (P. Kulabhusan et al., 2017). To date, the double antibody enzyme‐linked immunosorbent assay (ELISA) has been used to detect specific mAbs and capture polyclonal antibodies that can identify various epitopes to quantify antigens at the protein level (Y. Chang et al., 2016; Mecham, 2006). These have been proven to be a reliable and sensitive approach for detecting viral infections, and they might also be used to identify WSSV in shrimps (X. Tang et al., 2018). In the *Escherichia coli* medium, Murugan and Sankaran proposed a new ELISA approach for evaluating recombinant bacterial lipid change in proteins (Murugan and Sankaran, 2018). In that study, the most frequently generated white spot syndrome viral protein ICP11 was found as a new target in a novel ELISA method using a lipid‐modified protein. The scientists discovered that this approach was a predictive factor of antigen in contaminated shrimp tissues, with a DL of 250 pg for ICP11 protein and a dynamic range of 15–240 ng. The benefits of this test are that it has a low assay requirement, can be detected with the unaided eye and is the budget (Murugan and Sankaran, 2018) yet, the researchers did not mention if the detecting method is swift or takes a long time. Antibody‐based indirect immunofluorescence is another approach commonly employed in virological studies, though it is less sensitive than antibody‐based indirect immunofluorescence. In a study, indirect immunofluorescence's sensitivity and specificity were determined to be 29 and 96%, respectively, compared with PCR (Burgesser et al., 1999). As a result, the indirect immunofluorescence approach necessitates a huge density of viral particles in the host tissue, making virus detection at an early stage of infection impossible (Mott et al., 1997). Finally, despite the many advantages of immunological WSSV detection techniques outlined above (e.g., highly sensitive, specific and precise diagnosis) (Poulos et al., 2001), these approaches are often expensive, time consuming and need specialised equipment and expert workers making them unsuitable for use in the field (Poulos et al., 2001). Recently, a LFIA was developed using gold nanoparticles and a polyclonal antibody targeting WSSV VP28's main envelope protein. Through a simply specialised up‐gradation of the approach for selective virus detection, this method was reported to concurrently detect WSSV, IHHNV (infectious hypodermal and haematopoietic necrosis virus), MBV, HPV (human papillomavirus) and others. Furthermore, the approach is relatively less expensive (USD 3 per sample), and the farmer can execute the test without the assistance of an outside professional, reducing the farmers’ financial burden significantly. Consequently, the LFIA might provide practical assistance to farmers, fish farm operators and fisheries (P. K. Kulabhusan et al., 2017).

### 6.4 Molecular techniques

#### 6.4.1 Nested PCR

To improve the efficacy and accuracy of one‐step PCR, the findings of the first experiment can be amplified further in a nested second cycle of PCR (Peinado‐Guevara and lopez‐meyer, 2006). Since it includes two steps in the construction of the assay and typically requires the evaluation of reaction products on a gel, this sort of assay needs more hands‐on time than the others.

It is an audit method that is the gold standard recommended for use in WSSV inspections in shrimp. After following the first‐step PCR and second step of the (nested) PCR, which relies on two pairs of primers, the reading is performed by the electrophoresis gel method. The positive results in the first episode of the standard method criteria refer to the infection of the severe white spot virus, while when the positive results are obtained in the second period, it indicates that it is a latent or carrier infection (Nunan & Lightner, 1997). In addition to reading results under UV light or reading results with DNA strips, PCR output can also be evaluated with loop‐mediated isothermal amplification (LAMP), providing easy and convenient readings, especially in cases of non‐operational analysis, using a processing time of approximately 60 min (Kono et al., 2004). After the PCR output has been acquired from regular episodes, enter the LAMP technique, where the translation can be determined by the turbidity from the white sediment of magnesium pyrophosphate. If the gene is enlarged, white sediments are found. In addition, it is considered fluorescent paint using fluorescent substances such as SYBR Green I, filled after the lamp technique will change the default colour (orange) to green when viewed under UV light (302 nm) (Marshall and Johnsen, 2017).

#### 6.4.2 Quantitative real‐time PCR

It is a technique to increase the amount of genetic material, which can measure the amount of genetic material based on fluorescence, starting with mRNA and then turning it into cDNA using reverse transcriptase enzymes. Increased DNA content Studies in shrimp often used plasmids containing the genome part of the WSSV (U50923) for positive control and 10× diluted sensitivity tests to contain copies in the range of 2 × 10^5^ copies in standard curves and often using TaqMan and FAM‐BHQ1 probes (S. Durand and Lightner, 2002). In real‐time, PCR has a free fluorescence meter during the polymerase DNA reaction, which digests fluorescent labels to fall out of the primer, becoming a free fluorescent colour. Independent measurements of variations with genetic material content in WSSV diagnosis have applied the qPCR technique (Mendoza‐Cano and Sánchez‐Paz, 2013b; Y. L. Tsai et al., 2014). By relying on the reverse transcriptase enzyme called recombinase polymerase amplification (RPA), this reaction can be performed in one step, reducing the step for cDNA preparation, temperature is also used by RPA (32‐42°C), which is lower than that of conventional PCR. The number or synthesis of DNA represents a reaction that can happen even at normal temperature, so this method is a quick way to diagnose a disease (Lobato & O'Sullivan 2018), the qPCR technique was also developed, can be done at the same time. The duplex qPCR technique connects the two. By using only 2–20 DNA concentrations in the plasmid copies/reaction and 2 × 10^5^ copies/diagnosis of WSSV and IHHN, respectively, is considered high diagnostic sensitivity and specificity (K. F. Tang and Lightner, 2001).

#### 6.4.3 Fluorescent quantitative PCR

Another approach for quantifying DNA is quantitative fluorescence PCR (FQ‐PCR), where the result is decided by the concentration of DNA templates having fluorescent sequences (Bustin 2000). RT‐PCR is more expensive than this approach (Ririe et al., 1997); however, unlike dye‐based qPCR, any type of double‐strand DNA can produce fluorescence, making it less sensitive than TaqMan RT‐PCR (Ririe et al., 1997), TaqMan probes, on the other hand, are employed to improve the RT‐PCR sensitivity (Holland et al., 1991).

#### 6.4.4 Loop‐mediated isothermal amplification

The LAMP method is an innovative, sensitive and fast technology used in aquaculture to diagnose illness. The LAMP method developed a high sensitivity and diagnostic specificity test for identifying the WSSV. The WSSV genome DNA was used to build a set of four primers, two outside and two interior primers. The reaction time and temperatures were set at 1 h at 65°C. As contrasted to 10 fg using nested PCR, the DL of the LAMP approach was up to 1 fg (PCR). As a response, a standard LAMP technique was used to detect WSSV in the heart, stomach and lymphoid organs of diseased shrimp (Notomi et al., 2000). The LAMP process is an auto‐cycling strand displacement DNA synthesis that uses a high‐strand displacement DNA polymerase and a set of specialised primers to recognise six different patterns on target DNA. As a result, it is intended to amplify the target sequence selectively. The final LAMP outputs are a mix of diverse stem‐loop DNA lengths (Notomi et al., 2000). When the LAMP results are observed using agarose gel electrophoresis, numerous bands of various widths are visible up to the loading well.

#### 6.4.5 Pre‐amplification PCR

Researchers recently established a new pre‐amplification PCR approach, in which numerous short‐strand sequences were employed as adapters to ligate to the WSSV restriction pieces VP28, VP39 and VP108 via a link (X. Pan et al., 2017). Following that, using a universal primer, the ligated product is used as a template for pre‐amplification PCR. The pre‐amplification PCR considerably enriches the target DNA, and the pre‐amplified product is then used as a template for the particular PCR that completes the ultimate diagnosis. Such a non‐quantitative diagnosis methodology seems more accurate than nested PCR and can be used to diagnose WSSV at an early stage (X. Pan et al., 2017). The sensitivity of the PCR process, however, is greatly influenced by the method of DNA extraction, template concentration and amplicon size. A positive PCR test that finds a DNA fragment is also a poor predictor of viral opportunity (Cha and Thilly, 1993). Furthermore, in most situations, these tests necessitate long‐distance sample transfer from the area to the laboratories. And these tests usually come at a high cost, take a long time and necessitate highly skilled laboratory employees and devoted research facilities.

#### 6.4.6 Insulated isothermal PCR assays

Each iiPCR test employs a unique PCR format that only necessitates the usage of a simple source of heat to complete the PCR steps. It is used in a portable nucleic acid analyser created especially for iiPCR and can detect products automatically, allowing for WSSV identification at the pond's edge. This is comparable to real‐time qPCR in that it produces high‐sensitivity data instantly with little or no post‐reaction processes (Wilkes et al., 2018).

The test becomes more efficient and consumer friendly as product signals are automatically read, reducing human errors and data interpretation conflicts. iiPCR experiments take about 1.5 h to complete. Reagents and equipment for iiPCR assays are less expensive than those for real‐time qPCR tests. iiPCR tests, on the other hand, do not produce quantitative results, and the machines can only execute eight reactions per run. The OIE recently certified the I.Q. Plus WSSV Kit with POCKIT System, the only iiPCR‐based assay for WSSV, is appropriate for diagnosing WSSV.

#### 6.4.7 One‐step PCR assay

Traditional one‐step PCR procedures only amplify target DNA at a basic level, resulting in lower specificity than other PCR techniques. Gel electrophoresis, which comprises the separation of products through a gel of specific pore sizes in an electrical field (gel electrophoresis) and the staining and visual detection of product bands on the gel with the use of a fluorescent dye, is the most common method of product detection (Olive and Bean, 1999).

It usually takes 2–3 h to complete target amplification and product detection. In addition to being labour intensive and time consuming, these techniques carry the danger of cross‐contaminating amplicons in future assays, resulting in false‐positive results. On the other hand, experiments utilising this format are usually less expensive than other PCR assays.

#### 6.4.8 Duplex real‐time PCR

A duplex qPCR was standardised for WSSV and PstDV1 identification in clinical specimens employing valid and reliable primers and probes. Utilising the improved duplex qPCR settings, singleplex assays had specificities and sensitivities similar to those of multiplex tests, allowing the diagnosis of both singular and coinfections in infected white leg shrimp. Because such coinfections in shrimp are widespread, the concurrent identification of the WSSV and the PstDV1 is beneficial (Leal et al., 2014).

The analytical sensitivity of the duplex qPCR assay for WSSV and PstDV1 was comparable to that of singleplex assays. According to the researchers, this duplex qPCR technique discovered 10‐ and 627‐fold lower quantities of PstDV1 DNA than traditional duplex and multiplex PCR procedures (Leal et al., 2014). Moreover, the duplex qPCR test's WSSV DL was onefold lesser than multiplex PCR techniques and 10 copies lower than multiplex qPCR techniques. Several studies have shown that the standardised duplex qPCR approach outperforms other conventional and real‐time PCR‐based approaches. Furthermore, the standardised duplex qPCR assay's lower DL makes it a therapeutically relevant tool for identifying animals with low virus loads, such as those found in shrimp larvae and PL, minimising the risk of false‐negative results (Leal et al., 2014).

#### 6.4.9 Optimised PCR assay

Based on the nested PCR procedure described by Chu Fang Lo et al. (1996), a rapid PCR assay for the detection of WSSV was developed and outlined as the recommended PCR diagnostic assay in the Manual of Diagnostic Tests for Aquatic Animals published by the Office of International Epizootics. The second‐step primers utilised in the nested WSSV PCR were added to the optimised process. This modified assay is significantly faster and as sensitive as the recommended OIE procedure after lowering the annealing temperature and decreasing the cycling periods. The two‐step nested PCR methodology and a modified nested process were directly compared to the modified PCR test. The sensitivity of the published assay was tested using template dilutions of semi‐purified WSSV virions that had been quantitated using real‐time PCR for WSSV detection. The modified technique was used to examine various isolates to ensure that the assay could detect WSSV from various geographical regions.

#### 6.4.10 Fluorescence in situ hybridisation

**FIGURE 4:**
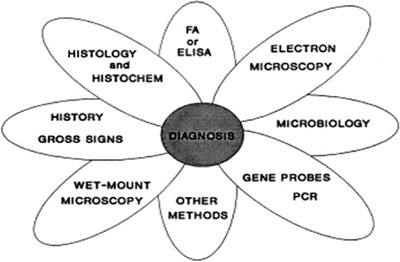
Schematic diagram of WSSV detection method

It is a method of detecting DNA that contains nucleoli. The tidying is specific to cells that show signs or suspected infection. DNA probes of approximately 50–100 nucleotides and peraluminous substances are attached. The probe will be able to capture the target nucleotide acid that contains nucleotides that match the DNA probes within the cells to translate the results into consideration. From the appearance of labelled colours on the probe, which requires the fluorescent microscope (Nunan & Lightner, 1997), with white colour with A WSSV virus infection, appears dark silver sediments. In addition to diagnosing viruses, fluorescence in situ hybridisation (FISH) is also used to study types of bacteria. The liver membranes, fillings and water are used to treat shrimp. FISH is a highly sensitive and cultured method. However, it is not suitable for diagnosis or diagnosis. As such, a PCR method has been developed to analyse abnormalities at a specific genetic level and is very sensitive (Bennour et al., 2016) (Figure 4 and Tables 2 and 3).

**TABLE 2:** Summary of PCR methods for WSSV detection

	PCR	Nested PCR	Real‐time qPCR	iiPCR
Specificity	+++	+++	+++	+++
Sensitivity	+	+++	+++	+++
Quantitation	Semi‐quantitation possible	Semi‐quantitation possible	Yes	No
Product detection	Visual	Visual	Automatic reading	Automatic reading
Post‐amplification	Yes	Yes	No	No
Cross contamination	+++	+++	+	+
Reaction time	++	+++	+	+

+++, high; ++, moderate; +, low.

**TABLE 3:** Primers used for detection of WSSV in shrimp

Methods	Sequence 5′–3′	Product size (bp)	Reference
Nested PCR	F1: ACTACTAACTTCAGCCTATCTAG R1: TAATGCGGGTGTAATGTTCTTACGA F2: GTAACTGCCCCTTCCATCTCCA R2: TACGGCAGCTGCTGGACCTTGT	1441 941	(S. Durand and Lightner, 2002)
Duplex qPCR	WSSV‐F: ′GCTGCCTTGCCGGAAATTA WSSV‐R; AGCCATGAAGAATGCCGTCTATCACACA Prob‐r: 6‐carboxyfluorescein (FAM) at the 5′ Prob‐q: BHQ1 at the 3′ IHHNV‐F: TAC TCC GGA CAC CCA ACC A IHHNV‐R: GGC TCT GGC AGC AAA GGT AA ^1^/Prob‐q: N,N,N′,N′‐tetramethyl‐6‐carboxyrhodamine (TAMRA) ^1^/Prob‐r: 6‐carboxyfluorescein (FAM) at the 5′	**–**	(Leal et al., 2014)
qPCR	F: ’GCTGCCTTGCCGGAAATTA R; AGCCATGAAGAATGCCGTCTATCACACA Prob‐r: 6‐carboxyfluorescein (FAM) at the 5′ Prob‐q: BHQ1 at the 3′	‐	(S. Durand and Lightner, 2002)
PCR	F: AATGGTCCCGTCCTCATCTCA R: GCTGCCTTGCCGGAAATT	71	(Y. L. Tsai et al., 2012)
RPA	F: CATGGATGAAAACCTCCGCATTCCTGTGAC R: CATCAGACTTTCCATTGCGGATCTTGATTTTG Prob: TGCTGAGGTTGGATCAGGCTACTTCAAGA(BHQ1‐dT) G(THF)C(FAM‐dT)GATGTGTCCTTTGAC (phosphate)	124	(Xiaoming et al., 2014)

## 7 TRANSMISSION

WSSV can spread horizontally and vertically (Leu et al., 2008; Prior et al., 2003; Soowannayan and Phanthura, 2011). There are three ways for the virus to spread among shrimp and other decapod crustaceans: (i) oral transmission via infected organisms or contaminated food; (ii) cutaneous transmission via infected organisms or contaminated food and (iii) cutaneous transmission via infected organisms or contaminated food. Furthermore, the virus can be transmitted through the consumption of affected tissues by parasitism and cannibalism, (ii) through waterborne transmission, the virus enters the body through the gills and external areas via direct contact with virus particles in water and (ii) through airborne transmission via the lungs and external areas by close contact with virus particles in water (Thuong, 2016) and (iii) through vertical transmission (via oocytes) from an infected broodstock to offspring (De Gryse et al., 2020; Sánchez‐Paz, 2010). According to various findings, the transmission rates in both *P. vannamei* and *P. monodon* species are similar. The authors believe, however, that the major impact of direct and indirect transmission rates might be different (Tuyen et al., 2014). Although pathogens have been found in oocytes in mature eggs they have not been found. This could signify that the virus‐infected oocytes are unable to develop into ovarian follicles (Y. G. Wang et al., 2000). The transportation of live and frozen uncooked shrimp can help transmit WSSV between different geographical locations (S. V. Durand et al., 2000), and broodstock imported from different countries and regions (Stentiford et al., 2009). Environmental factors such as temperature, fluctuation in salinity and pH have been identified as major stressors that can influence WSSV infection transmission and outbreaks, according to several studies (Van Thuong et al., 2016; Yu et al., 2006). Several studies in Thailand were undertaken to assess the impact of weather conditions on WSD infection, and the results showed a higher incident ratio of disease at the time of mean ambient temperature fluctuating from 24.5 to 27.23°C, based on the data gathered (Gronski, 2000; Nakorn et al., 2017; Piamsomboon et al., 2016).

## 8 CLINICAL SYMPTOMS AND ESSENTIAL NATURE OF WSSV

**FIGURE 5:**
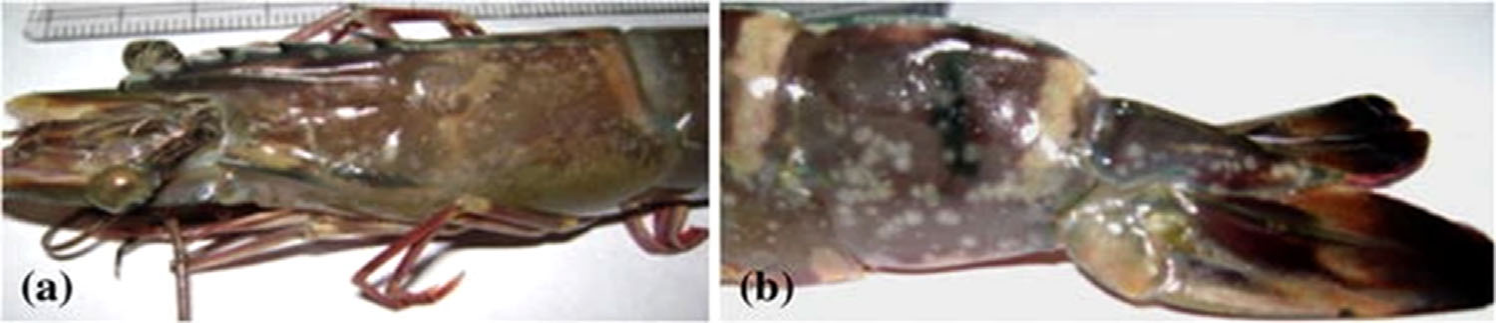
Infected with the white spot syndrome virus, symptoms of *Penaeus monodon* on the carapace (a) and last abdominal segment (b)

WSSV takes a long period to manifest, and if it does, infected animals die between 3 and 8 days, leading to a higher fatality rate (Corbel et al., 2001). WSSV‐infected shrimp assemble at the pond's side in the natural environment and display pathological signs 1 or 2 days before any death happens. Within 10 days of the commencement of the disease, cumulative death may exceed 100% (J. M. Lotz and Soto, 2002). Juvenile shrimp of all ages and sizes are pathogenic in grow‐out ponds, but significant mortality occurs either 1 or 2 months upon stocking (Kasornchandra et al., 1998; Mishra and Shekhar, 2005). The most common sign of WSSV infection is the appearance of circular white spots or patches of 0.5–3.0 mm in diameter, especially noticeable in the cuticle of the cephalothorax and tail section (Kim et al., 1998; Chu Fang Lo et al., 1996). White to reddish‐brown/reddish/pinkish/ to discoloration are some of the characteristics of WSSV infection; over the head and carapace, reduced appetite, congregation near the embarkation and other symptoms that are not unique to bacterial white spot syndrome (Corbel et al., 2001; Y. T. Wang et al., 2002). Although the specific process of white spot creation is unknown, it is conceivable that WSSV infection causes integument malfunction, leading to calcium salt accumulation inside the cuticle and the production of white spots (Y. G. Wang et al., 2000). This disease also causes a red coloration of the skin and appendages due to the expansion of chromatophores (Alfaro et al., 2021; W. Zhan et al., 2000), fewer feed intake (Nadala et al., 1998), reduced preening and poor reaction to a stimulus (Durand et al., 1997), loose cuticle (Zhou, 1999), enlargement of branchiostegites due to fluid buildup (W. Zhan et al., 2000) and thinning and delayed clotting of haemolymph (Q. Wang et al., 2001). Infected cells’ hypertrophied nuclei have eosinophilic to increasingly basophilic inclusion masses (Afsharnasab et al., 2014). Infected nuclei become increasingly basophilic and expand over time (Takahashi et al., 2000; Y.‐C. Wang et al., 1998). During the late stages of infection, karyorrhexis and cellular disintegration might occur, resulting in vacuolisation in necrotic regions (Karunasagar et al., 1997; Kasornchandra et al., 1998) (Figure 5).

## 9 WSSV IMPACTS ON SHRIMP FARMING

WSSV is currently the biggest serious threat to Asia's shrimp farming business and has been for some time. This is a very virulent disease that can infect a broad host range. Moreover, profits were lost due to secondary losses in hatchery, feed and packing factory capabilities, among other things.

Although there is little information on the existence and consequences of WSSV in wild shrimp communities in afflicted areas, it is predominantly located in Asia and Latin America (Walker & Mohan, 2009). The cuticular epithelium, connective, neural, muscular, lymphoid and haematopoietic tissues are among the ectodermal and mesodermal tissues infected by WSSV. The stomach, gills, antennal gland, heart and eyes are all badly affected by the virus. In the final stages of infection, these organs are damaged, and several cells are lysed. Hepatopancreas of shrimp turns crimson and white spots (inclusions) with a diameter of 1–2mm appear on their carapace, limbs and interior body parts (Kv et al., 2005). They also exhibit sluggish behaviour, with cumulative mortality reaching 100% between 2 and 7 days of illness. Although it has been increasingly evident since the late 1990s that the occurrence of WSSV in an aquatic system does not invariably portend tragedy. Environmental changes, likely related to osmotic stress via salt concentrations or hardness, or abrupt temperature fluctuations, have generated outbreaks from latent *P. monodon* carriers in Thailand (de la Vega et al., 2007). Likewise, in Latin and North America, temperature fluctuations were shown to cause infected *P. vannamei* to die. Nevertheless, there have already been various claims about the ambient temperature that have been disclosed to limit WSSV death rate at 18 or 22°C and induce 100% fatalities at 32°C in the United States, but induce mortality at less than 30°C and prevent it at significantly larger than 30°C in Ecuador (Verbruggen et al., 2016). Consequently, 3–4 years of gene evaluation work on domestic *P. vannamei* stocks (selection of shrimp surviving WSSV outbreaks) appears to have improved WSSV resistance in Ecuador. Since the outbreak of the WSSV in 1999, in Central and South America, the cultural sectors for *P. vannamei* have been steadily rebuilding. Ecuador, for example, exported 115,000 metric tonnes in 1998, but only 38,000 metric tonnes in 2000 following the arrival of WSSV in 1999. Ecuador has since recovered, with an estimated export of 50,000 metric tons in 2003 (Cuéllar‐Anjel et al., 2012).

## 10 WSSV VIABILITY in POND SEDIMENTS

WSSV is found in large quantities in shrimp pond sediment. The most significant metric in the transmission of WSD in newly stocked ponds is WSSV viability in sediment. The physicochemical properties of the sediment influence the viability and infectivity of WSSV. WSSV could last for up to 19 days after being exposed to the sun (De Gryse et al., 2020). Under actual field conditions, WSSV could remain infectious in pond sediments for up to 32 days after harvest. The significant difference in WSSV viability in pond sediment could be related to the pond sediment's early viral load, the physical and chemical features of the pond sediment and the incidence of the disease epidemic during the previous crop, which contributed to this. Infectivity bioassays show that acute to subacute WSSV disease in shrimp PL can be caused by fresh and 7‐day post‐harvest sediment samples, with mortalities appearing within 8–15 days. Sediment samples taken 14–32 days after harvest can cause chronic WSSV infection in shrimp PL, with mortality occurring between 18 and 25 days. An earlier study found that WSSV DNA could be stored in the soil for up to 10 months under experimental conditions (Natividad et al., 2008).

Virus persistence in the environment is influenced by various physical, chemical and biological factors (C. P. Gerba, 2005). Temperature, daylight, osmotic pressure, inorganic chemicals, microbial antagonism, viral sorption state and virus type all influence virus survival in sediment. The number and kind of organic matter, as well as pH, moisture content and electrical conductivity, are all factors to consider, among other chemical components of sediment, which may influence the persistence of viruses in soil (Bosch et al., 2007). (Sobsey et al., 1986) compared hepatitis A virus inactivation in five different soil types, including clay, clay loam, loamy sand, sandy and organic muck, the soil clay had the best survival rate. Clay has a stronger affinity for viral particles due to adsorption and it protects the virus from virucidal chemicals and thermal breakdown. The biological composition of a virus can have a significant impact on its survival and persistence in the environment. Heat, desiccation and acidic environments make enclosed viruses more vulnerable than non‐enveloped viruses, which can tolerate these conditions. The enveloped virus, like WSSV, is likely to be stabilised and protected in the aquatic environment by dissolved, colloidal and solid organic matter. According to previous research, the WSSV is stable at a pH of around 8, and it becomes utterly non‐infectious at pH levels as low as one and as high as 13 (Durand et al. 1997). By controlling the virus's adsorption on soil particles in sediments, pH can indirectly influence virus viability (Yates et al., 1987). Nonetheless, it is well understood that the kind and amount of saline in the ecosystem substantially impact viral transmission at the bottom. According to previous research, the WSSV remains stable at salinity levels ranging from 0 to 10% (Durand et al., 1997). Viruses are desorption from soil particles during rainfall due to a decrease in soil salt concentration. In soil, humic acid and fulvic acid can inhibit virus pathogenicity, limit adsorption to organic matter by battling for adsorbates and even function as an eluting agent, causing virus desorption (C. P. Gerba, 2005). Most research on the survivability of several viruses in wet and dry soil, including poliovirus, enterovirus, coxsackievirus and echovirus, discovered a positive link between virus survival and moisture levels (C. Gerba, 1999). The viral nucleic acid is degraded in moist soil before it is released from the virus capsid coat protein. The virus loses its infectivity in soil under dry conditions due to degeneration of the capsid protein coat, but the nucleic acid can be restored undamaged from the sediment (Mayer et al., 2015). Temperature, depth of the soil layer and distance from a nearby river basin are all said to impact soil moisture content. Numerous investigations on the influence of temperature on poliovirus survival have discovered that as the temperature increases, so does the inactivation rate (Cliver, 2009). Denaturation of the viral capsid protein may cause viral inactivation at higher temperatures (Yan et al., 2004). The temperature indirectly affects virus survival by lowering the moisture content of the soil, particularly in the upper layer. The viral particles in the lower layers of the soil, on the other hand, remain viable due to the protection from sunlight provided by the top layer of soil. The chemical disinfectant sodium hypochlorite is commonly used in shrimp farms to disinfect water, soil and equipment. It is an effective disinfectant for pond soil decontamination. After 10 min of treatment with sodium hypochlorite at a concentration of 100 ppm, WSSV becomes non‐infectious (Oseko et al., 2006). According to another study, shrimp ponds rinsed with 100 ppm bleaching powder were infected with WSSV‐positive invertebrates 20 days post disinfection (Burge, 1981). Disinfection of soil in real‐world farming conditions appears to be extremely difficult. The treatment's efficacy would be determined by the amount of active chlorine in the commercial preparatory work.

## 11 ENVIRONMENTAL IMPACT of WSSV

Multicellular organisms’ typical physiological processes necessitate a reasonably steady internal redox status. External variables, for instance, mycotoxin and heavy metal stress, on the other hand, might cause an internal redox state imbalance, potentially leading to oxidative stress. Reactive oxygen and nitrogen species (ROS and RNS) are produced as a result of oxidative stress, and they damage DNA and proteins, impairing normal cellular activity. As a result, organisms have devised several ways to counteract such harm. To eliminate ROS and RNS, downstream enzymes such as superoxide dismutase (SOD) and glutathione reductase are often utilised, as well as upstream signalling recognition and signalling transduction molecules. Several enzymes involved in redox reactions have been researched in shrimp, with SOD being the most well researched. MnSOD and Cu/ZnSOD have been identified in shrimp. FeSOD had yet to be discovered (Mai et al., 2014;Yao et al., 2004). Shrimp are commonly exposed to heavy metals. The effects of heavy metal pollution on shrimp, as well as the mechanisms of heavy metal resistance, have mostly focused on metallothionein (MT). The sulfhydryl group of MT aggressively chelates harmful metals and expels them from the body, making it a metal‐binding protein high in cysteine (Ecker et al., 1987). Environmental stress has a well‐documented impact on macroscopic markers of physiological status and immunological response. Strong or long‐term environmental stressors have a deleterious influence on shrimp's innate immunity in general, and different environmental conditions have somewhat different effects. The intestinal cell wall is the initial line of defence versus foreign matter invasion in *L. vannamei* (Vidal et al., 2001). The integrity of the cellular wall structure, the makeup of intestinal bacteria and the immunological components in the intestinal mucus all play a role in the intestinal wall's efficacy. However, in *L. vannamei*, ammonia‐nitrogen exposure damages the intestinal mucosa and disrupts the makeup of intestinal microorganisms, resulting in a reduction in intestinal immunological function (Duan et al., 2018). In *Fenneropenaeus indicus*, the pH of cultured water is directly connected to total hemocyte count (THC) and total hemocyte pPO (Sharma S R et al., 2009). Plasma RB activity, malondialdehyde levels and hemocyte DNA damage levels all rose significantly in *L. vannamei* exposed to cold stress (12°C), although plasma THC, total protein content and osmotic pressure were all reduced (Qiu et al., 2011). Changes in salinity within a particular range (22–14) had little effect on *L. vannamei* THC, but significantly reduced pO activity, which resulted in enhanced WSSV replication (Yu et al., 2006). The immune system of shrimp has been demonstrated to be weakened by water pH, temperature and other physical and chemical variables. On the other hand, environmental stress of the proper level has been demonstrated in certain experiments to generate immune‐like effects in shrimp, offering a degree of protection. Heat stress (33°C) during the early stages of WSSV infection was also found to boost WSSV resistance and reduce cumulative shrimp mortality in *L. vannamei* (M. Rahman et al., 2007; Rahman et al., 2006). Indeed, both cold and heat shocks reduced the cumulative mortality of WSSV‐infected shrimp (Yuan et al., 2017). Furthermore, injection with ‐glucan raised ROS and produced oxidative stress in WSSV‐infected shrimp, but substantially decreased WSSV viral load; yet, the cumulative death rate of the infected shrimp remained greater than the control category (Thitamadee et al., 2014). Some effects of environmental conditions on viral illness in shrimp have been observed since the 1990s. Nitrogen content, nitrite nitrogen and saltiness of aquatic water all affected shrimp MBV infection in *P. monodon*, according to (G. Li et al., 2001). MBV was eventually superseded as the most dangerous virus to Shrimp by WSSV. Every year since the first breakout of WSS in 1992, it has resulted in significant losses of cultivated shrimp. Shrimp with WSSV is still frequent in shrimp farms today. Shrimp WSS has been proven to be directly related to acute factors in both production practice and laboratory study (Al‐Bahry et al., 2011; Y. Chen et al., 2010; J. G. He et al., 2000) (in Chinese), and in recent years, its mechanism has been increasingly elucidated. To better understand the processes of environmentally induced WSS in shrimp, researchers looked at the impacts of a variety of environmental stresses on WSSV infection. It has been demonstrated that heat stress (32°C) enhanced HSP70 transcription, and that stressed shrimp used RNAi to lower HSP70 expression. At the same temperature, the shrimp were more vulnerable to WSSV than the treatment group (Y.‐R. Lin et al., 2011). Scientists previously demonstrated that increasing or decreasing the water temperature correctly improved *L. vannamei* resistance to WSSV (Yuan et al., 2017). Shrimp are very poisonous to molecular ammonia nitrogen. Shrimp exposed to ammonia nitrogen had much more WSSV copies in their hepatopancreas, hemolymph and swimming feet than control shrimp (Mohamed Fouzi et al., 2010). Shrimp's capacity to combat WSSV infections was decreased by osmotic stress. WSSV replication in shrimp is inhibited mostly by ROS produced under oxidative stress (H.‐H. He et al., 2017; Su et al., 2014; Thitamadee et al., 2014). Shrimps have also been found to be more sensitive to WSSV under hypoxic settings, probably due to anoxic circumstances releasing fewer ROS. As a result, most ecological stresses impair shrimp susceptibility to WSSV (Lehmann et al., 2016)

## 12 ECONOMIC CONSEQUENCES of the WSSV VIRUS

WSSV has a significant economic impact on aquaculture around the world. After the first epidemic in 1992, production decreased by more than 70%, resulting in a 3‐year production loss of more than US$2 billion in China. Before the WSSV outbreak, Thailand's yearly production rate was around 34,000 tonnes per year, but by 1994, it had dropped to 265,000 tonnes, worth US$1.6 billion. Indonesia followed a similar pattern, with production gradually increasing to 17,000 tonnes per year from 1985 to 1991 until the WSSV outbreak. Due to the WSSV, their production fell in 1992, resulting in projected output losses of almost $1 billion over the next 10 years. In Ecuador, an outbreak occurred in 1999, and production fell by over 60% in 2 years, resulting in losses of over $1 billion between 1998 and 2001 (Ghosh, 2014). A similar situation was recorded in Panama and Peru, where production decreased by 90%, resulting in losses of over US$100 million and US$70 million over 3 years, respectively. Since 1992, India's shrimp aquaculture revenue has decreased to a loss of INR 2700 million (Xu et al., 2007). In 1994, the illness expanded to Bangladesh's southwest area, damaging roughly 90% of large shrimp farms and creating a 20% decline in national shrimp production. As a result, Bangladesh's shrimp exports fell from 25,742 tonnes in 1997–1998 to 18,630 tonnes (Debnath et al., 2014).

## 13 MANAGEMENT TECHNIQUES FOR WSSV

At first, avoid purchasing nauplii or PLs from sources that may or may not be infected with the virus. When used on eggs, nauplii and PLs, iodine and water washes will likely eradicate the virus. This procedure must be followed consistently. Incubation facilities should maintain high biosecurity norms and inspect every animal batch. A hatchery should be constructed to prevent the infection from being presented in the ocean. To diagnose and screen WSSV, utilise the most sensitive and dependable demonstrative tests accessible (Powell et al., 2006). Tissue lesions will be studied using DNA‐based tools such as PCR and in situ hybridisation (ISH). At least twice during the production cycle, sample hatcheries and keep samples for subsequent testing (3 weeks post shipping). Only purchase animals that have been PCR checked and stress tested (Sterchi, 2008). It's critical to start with no or a very low viral load. It has been discovered that stressing PLs with formalin helps to screen out weaker animals, yet it is never a good idea to stock PLs that are known to be infected with the virus. It has been noticed that the virus in *P. japonicus* may not show pathogenicity until after PL6, necessitating the screening of later‐stage animals. Reduce the shrimp's stress as much as possible. You can do it in a few different ways and cannot do it in many of them. Some of the things you can do include:

1. Before stocking, increase acclimation times.
2. Increase stress tolerance with non‐specific immune stimulants (NSIS) and supplemented mineral and vitamin diets.
3. Consider stocking during periods of the year when you know the environment will not be subjected to extremes in temperature and salinity. It has been claimed that these pressures can trigger an epizootic in a virus‐infected population.
4. Maintain a high‐quality diet and employ NSIS throughout your life cycle.
5. If conceivable, stock at lower densities.
6. Keep an eye out for WSSV‐carrying vectors in the ponds and take steps to eliminate them.
7. Before stocking, collect phytoplankton and zooplankton from the pond and test for WSSV using PCR. Positive ponds should be avoided at all costs.
8. Sample your ponds regularly. Ascertain that sick and dying animals are routinely collected by PCR and histology, as well as any unexpected patterns of death. If you can, harvest the shrimp at the first sign of a problem.

### 13.1 Biosecurity for shrimp farming

**FIGURE 6:**
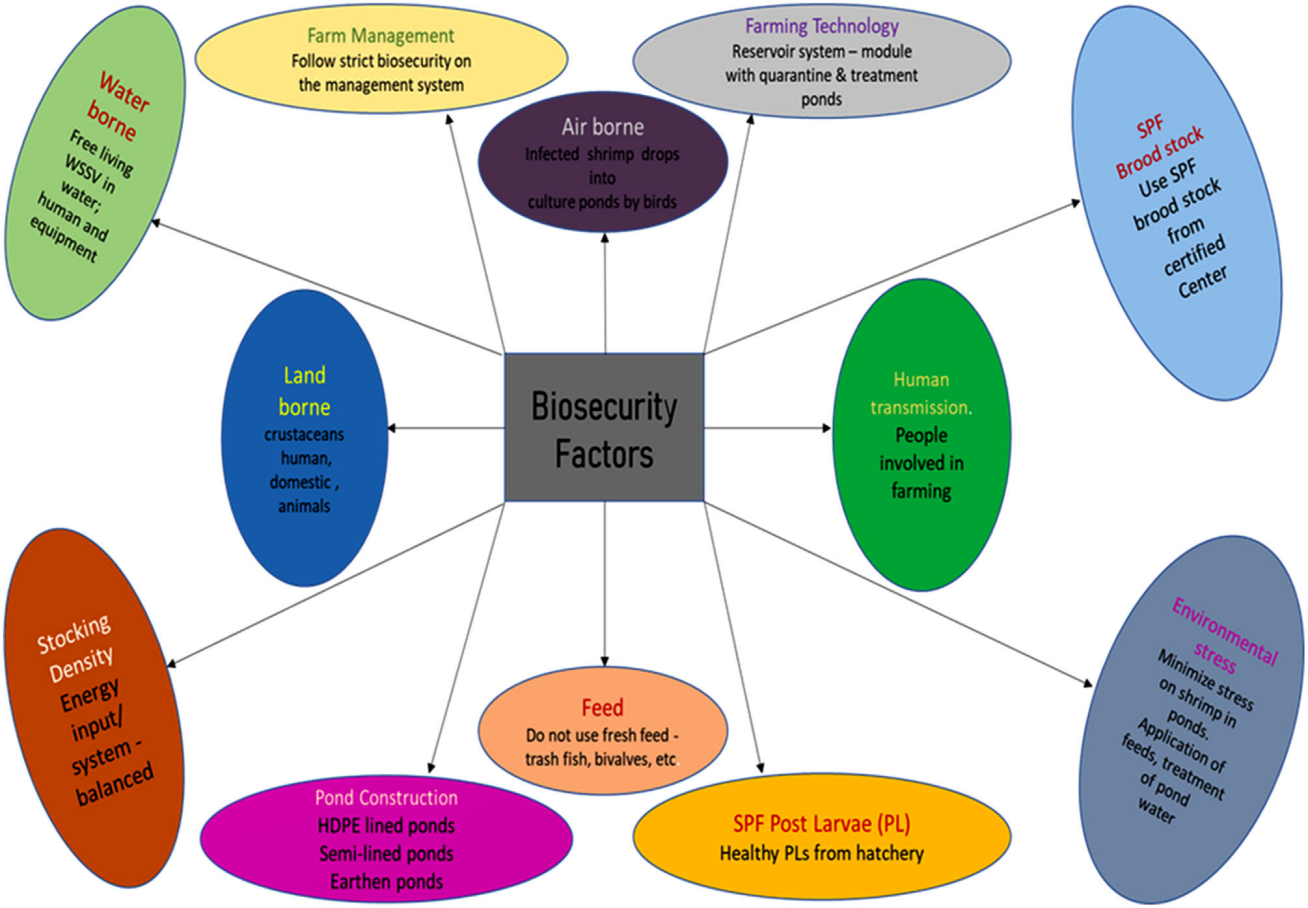
Farm biosecurity factors

**FIGURE 7:**
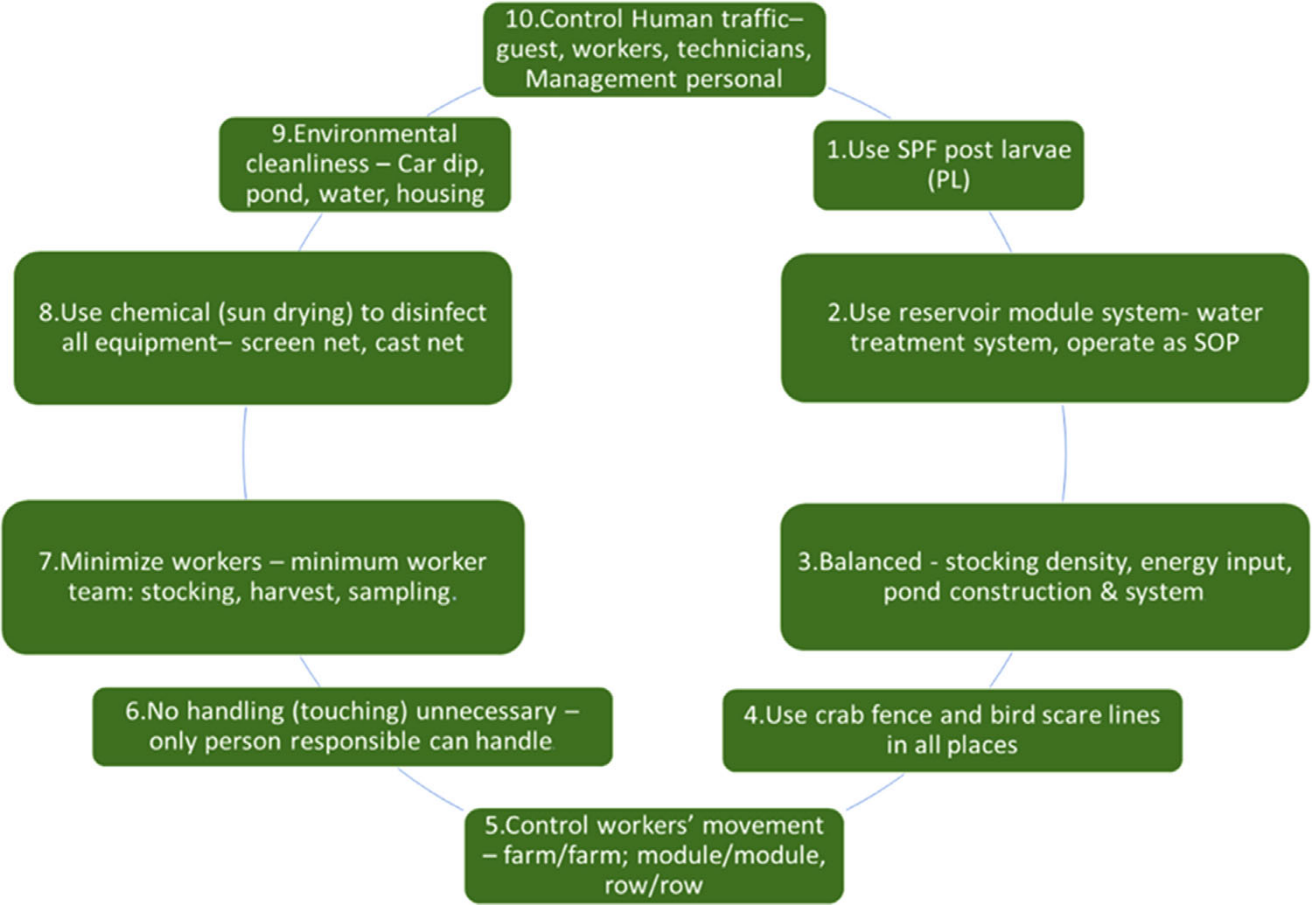
Farm biosecurity – implementation

Biosecurity is more accessible to deploy in small, intensive and regulated farming systems than in large‐scale outdoor activities. In the shrimp industry, biosecurity measures are divided into two categories: excluding pathogens and eradicating infections when they are present.

(D. Lightner, 2003) highlighted measures to keep diseases out of stock (i.e., PL and broodstock), including quarantine and SPF, certified stocks and limiting live and frozen shrimp imports. Removing diseases from hatcheries and farms was advised by excluding vectors and external sources of pollution and protecting against internal cross‐contamination (Figures 6 and 7).

### 13.2 Prevention

The methods of prevention are similar to those used in the case of TSV. Before importing live or frozen shrimp from disease‐infected to disease‐free zones, PCR testing needs to be performed on all live and frozen shrimp (Dhar et al., 2004). Before breeding, PCR testing of broodstock should be performed. Before stocking into ponds, PL should also be PCR screened, resulting in an increased percentage of good harvests. Although PCR is not a perfect way of diagnosing white spot virus, this is currently the most accurate diagnostic procedure. Vertical transmission of WSSV from infected broodstock to larval stages can also be prevented by washing and disinfecting eggs and nauplii. Fresh crab and other crustaceans should never be fed to broodstock (P. Liu et al., 2000). Polyculture strategies involving somewhat carnivorous fish species (Tilapia) have also been demonstrated to reduce WSSV pathogenicity in the farm areas, so the fish can consume infectious vectors before contacting live shrimp. Because this virus can only survive 3–4 days in water, preventing transmission can be as simple as disinfecting the water used for adjustments and using precise filtration (H. Liu et al., 2019). Formalin doses of 70 ppm have been shown to prevent transmission while causing no damage to shrimp. Furthermore, all wastewater from farming or processing operations with the potential for WSSV infections should be treated before discharge (e.g., with formalin or chlorine). PCR and probes for dot‐blot and ISH tests can detect WSSV. The presence of characteristic white spots can likewise be utilised to analyse it visually (albeit these are not generally present in infected animals) (McColl et al., 2004). The presence of huge numbers of Cowdrey A‐type nuclear inclusions and hypertrophied nuclei in H&E‐stained sectioned tissues, or simply fixing and staining gill tissue and studying it under a microscope, can be used to confirm WSSV (especially for asymptomatic carriers) (Pazir et al., 2012).

## 14 PRODUCTION OF SPF SHRIMP

The collection of apparently healthy wild stock from areas with low disease frequency, followed by individual primary quarantine, where the shrimp can be individually checked for specific infections and contaminated individuals eliminated, are significant components of an SPF procedure(Barman et al., 2012). The shrimp are then moved to a secondary quarantine facility, which is raised to broodstock size and constantly checked. Healthy broodstock is subsequently sent to the breeding centre, where many families from various sources are produced (Barman et al., 2012). The larvae are then raised in biosecure hatcheries from the selected families. Any diseased stock discovered through continuous monitoring is discarded right away. The fundamental steps for developing SPR lines of broodstock *P. monodon* are similar to those for the SPF program. A more rigorous genetic selection approach, including a more significant number of families, is necessary to select for desired features. Whatever program is chosen, developing SPF and/or SPR lines of *P. monodon* should be viewed as a long‐term commitment (D. Lightner, 2011). It requires consistent authority over all components of culture, exceptionally gifted logical individuals, the most significant levels of discipline and collaboration, specialised staff training and progressing research centre examination.

## 15 ADVANTAGES OF SPF

SPF animals give some certainty to a nation presenting a species interestingly that the imported animals will not spread the recorded diseases to local species. SPF stocks, on the other hand, may include unidentified infections, which should be considered because they can constitute a concern when the animals are stressed (Claydon et al., 2010). Biosecurity techniques are used to combat the possibility of disease outbreaks in shrimp culture. The principle point of biosecurity frameworks is to keep illnesses from spreading and to aid in their elimination if they do. Because the specific pathogen can be eradicated and contamination minimised, the particular pathogen‐free stock is one of the most important components in any biosecurity system (Aarattuthodiyil and Wise, 2017). Because the specified interfering infections can be ruled out, SPF animals are incredibly beneficial for basic and applied scientific research, particularly immunological studies and vaccination trials. SPF animals are also required for other bioassays; for example, a study of shrimp virus infections requires pathogen‐free animals because the shrimp cell line is unavailable (Barman et al., 2012).

## 16 LIMITATIONS OF SPF SHRIMP

All microorganisms representing a critical danger should be identified and physically removed from the stocking area. It should be noted, however, that the shrimp could in any case, be infected with a microbe, not on the list. SPF shrimp offspring are not SPF unless grown and kept in a biosecure SPF facility (Washim et al., 2019; Wyban, 2010). They can no longer be classified as SPF after they leave the institution. Managing *P. monodon* must be more troublesome than working with *L. vannamei* (V. Sedhuraman et al., 2014). Due to the difficulty of maturing *P. monodon* in captivity, a significantly longer holding period is needed before they arrive at a feasible size (D. V. Lightner, 2005).

## 17 SPR SHRIMP

SPR stands for SPR and is another frequently misunderstood acronym. It refers to a shrimp genetic feature that offers some resistance to a particular virus. SPR shrimp are usually the outcome of a specialised breeding effort to increase viral resistance. SPF and SPR are two distinct qualities. SPR shrimp may not all be SPF shrimp and vice versa. SPR shrimp strains, on the other hand, do not have to be SPF (Alday‐Sanz, 2018). Because of their superior presentation in broodstock as well as in grow‐out ponds, and frequent monitoring of disease outbreaks with the quarantine process for SPR, *P. vannamei* is currently almost exclusively used in Latin America. According to a recent FAO survey, there were over a hundred maturation units delivering 15 billion nauplii each 30 days and loading approximately 400 hatcheries, largely of SPR shrimp mostly in Ecuador and Mexico (Ghanaatian, 2015). TSV can create severe mortalities in *P. vannamei‐*stocked farms and easily spread across ponds by insect or avian vectors. As a result, the introduction of strains against TSV, with the advancement of biosecurity applications to prevent infections from other viruses, might significantly boost the production of the *P. vannamei* in Asian countries. The United States industry has embraced such a protocol, resulting in a 50% annual growth rate during the previous 3 years (Thongrak et al., 1997). Recently, some work has been done on generating a *P. chinensis* strain with SPR for WSSV. Ponds filled with PL produced by WSSV survivors, pandemic saw an increase in survival rates from 0–0.8% to 12–45%, whereas laboratory test examinations demonstrated 30–60% enhancements in overall survival for third and fourth‐generation winners. Both control and select animals exhibited a 60% infection rate with WSSV, according to PCR testing, proving that this was related to resistance (Kong et al., 2003). It is indeed possible that WSSV‐resistant *P. vannamei* strains will emerge. Even though WSSV is still the most severe disease risk in the Asian shrimp aquaculture industry, it could boost the sector itself well. The recent discovery of multiple molecular markers (particularly microsatellites) linked to immune function and growth, as well as potential advancements in quantitative genetics to shrimp breeding, suggest that selecting fast‐growing, disease‐resistant variants could become much more productive shortly. This could also provide insight into invertebrate antiviral immunity, which is currently unknown. In *P. stylirostris*, such disease‐related indicators have already been discovered (Hizer et al., 2002).

## 18 RECOMMENDATION TO CONTROL WSSV AT THE FARMING LEVEL

The movement of infected PLs mostly determines the viruses and other diseases’ mobility. As a result, if this can be stopped, the virus's transmission will be slowed. Numerous nations have found a way to accomplish this, however, the truth will surface eventually about how fruitful they will be. The virus appears to be here to stay once it takes a footing. Luckily, as with all other viral infections like BP, IHHN, TSV and MBV, the infection's effect will blur over a long period (Mani et al., 2016). The Thais adapt to the infection with a range of devices, some of which will be valuable for *P. vannamei* (C. Lin, 2020). However, it is critical to grasp the variations between the two types of shrimp cultivation, as well as the difficulties that come with utilising the same methods to try to limit the virus's presence. Pesticides should be used to kill the vectors before replenishing the ponds, according to the advice given to and received by Thai farmers (Szuster, 2006). Typically, a powerful insecticide is added to the water to kill any crustaceans or other virus‐carrying vectors that may be present in the pond. This is impossible to achieve on the scale required across most of America's regions. The expenses would be prohibitively exorbitant, and the massive volumes of pesticides that would have to be poured into the environment would be unwelcome. This should be avoided at all costs and only used as a last option. One potential method for removing some vectors is to filter incoming water entering the pond (Newman, 2019). Because of the high demand for water in the Americas, conventional filters might be hazardous. This demand, however, can be controlled by adding only a tiny amount of water to the system. Filters that catch everything using plant fibres in combination with serial mesh filtration could be effective. Using mesh filters with a pore size of 250 microns or less, as well as small mesh bags, can be beneficial (Taslihan et al., 2021).

Avoiding the exchange of water is another suggestion. Recent outbreaks of WSSV in South Carolina, USA, appear to be linked to the addition of water to ponds, therefore there appears to be some weight to this theory (Boyd et al., 2020). It is unclear if this stressed the shrimp and caused an outbreak, or whether it transferred vectors and viruses into the ponds. Aeration is used in intensive systems but not in semi‐intensive systems. In ponds where oxygen levels cannot be regulated without water exchange, the capacity to mechanically aerate the water without resorting to water exchange could be quite valuable (Hopkins et al., 1993). Animals that have been PCR positive for the infection are not bought. Screening supplied animals for the presence of the infection has been accounted to be a successful methodology for following the disease's status in the population in certain conditions. This can be used to time harvests and prevent problems from spreading to adjacent ponds and/or neighbours.

## 19 CONCLUSION AND SUGGESTIONS

WSSV remains a crucial disease in shrimp production and a big danger to the global shrimp business. WSD is attracting a lot of scientific attention these days, owing to the disease's economic implications. Currently, research is being conducted to achieve a remarkable comprehension of the molecular and pathophysiology of WSSV, as well as the proper treatment and prevention of the virus. Nevertheless, additional research should be done in places with more resistant host species to comprehend their function against disease effects, as these insights could be helpful in finding a cure for WSD. SPR shrimps are preferred over SPF animals for cultivation in locations where the disease is endemic. As a result, more research should be done in the breeding program to develop SPR animals which are resistant to WSSV. Notably, various studies in the domain of WSSV immunisation have been undertaken, although their field application has not yet succeeded. As a result, intervention and development of vaccines for commercial uses can help to minimise the risks. On the other hand, advanced techniques such as shrimp farming in recirculating aquaculture systems and polyculture could be the potential strategies for WSD regulation.

## AUTHOR CONTRIBUTIONS

*Conceptualisation, supervision, writing – original draft and writing – review and editing*: Sk Injamamul Islam; *Conceptualisation and writing – review and editing*: Moslema Jahan Mou. *Conceptualisation and writing – review and editing*: Saloa Sanjida.

## CONFLICT OF INTEREST

The authors declare that they have no competing interests.

### ETHICS STATEMENT

Ethics statement is not applicable as no sample collection or questionnaires was conducted.

### PEER REVIEW

The peer review history for this article is available at https://publons.com/publon/10.1002/vms3.979.

## Data Availability

No.
